# Global, regional and national burden of disease attributable to 19 selected occupational risk factors for 183 countries, 2000–2016: A systematic analysis from the WHO/ILO Joint Estimates of the Work-related Burden of Disease and Injury

**DOI:** 10.5271/sjweh.4001

**Published:** 2022-02-25

**Authors:** Frank Pega, Halim Hamzaoui, Bálint Náfrádi, Natalie C Momen

**Affiliations:** 1 Department of Environment, Climate Change and Health, World Health Organization, Geneva, Switzerland; 2 Labor Administration, Labor Inspection and Occupational Safety and Health Branch, International Labor Organization, Geneva, Switzerland

**Keywords:** occupational epidemiology, work-related disease, work-related injury, working hour

## Abstract

**Objectives:**

We provide a brief introduction to the objectives, data, methods and results of the World Health Organization (WHO)/International Labor Organization (ILO) Joint Estimates of the Work-related Burden of Disease and Injury (WHO/ILO Joint Estimates), which estimated the burden attributable to 19 selected occupational risk factors.

**Methods:**

The WHO/ILO Joint Estimates were produced within the global Comparative Risk Assessment framework, which attributes the burden of one specific health outcome (ie, disease/injury) to a specific occupational risk factor. For 39 established occupational risk factor-health outcome pairs, estimates are produced using population attributable fractions (PAF) from recent burden of disease estimates. For two additional pairs, PAF are calculated from new databases of exposure and risk ratios produced in WHO/ILO systematic reviews. Attributable disease burdens were estimated by applying the PAF to total disease burdens.

**Results:**

Globally in 2016, it is estimated that 1.88 [95% uncertainty range (UR) 1.84–1.92] million deaths and 89.72 (95% UR 88.61–90.83) million disability-adjusted life years were attributable to the 19 selected occupational risk factors and their health outcomes. A disproportionately large work-related burden of disease is observed in the WHO African Region (for disability-adjusted life years), South-East Asia Region, and Western Pacific Region (for deaths), males and older age groups.

**Conclusions:**

The WHO/ILO Joint Estimates can be used for global monitoring of exposure to occupational risk factors and work-related burden of disease and to identify, plan, cost, implement and evaluate policies, programs and actions to prevent exposure to occupational risk factors and their associated burden.

Despite a long history of productive interagency collaboration, the World Health Organization (WHO) and the International Labor Organization (ILO) have, until recently, produced separate estimates on work-related burden of disease. Their different methodologies have yielded different results. The two United Nations (UN) specialized agencies have been asked by Member States to harmonize their estimates. Additionally, the Sustainable Development Goals (SDGs) and the UN 2030 Agenda (1) call for partnerships for development and improved policy coherence. In response, an agreement was made in 2016 between WHO and ILO to develop a joint estimation methodology and produce the most comprehensive set of official estimates of work-related burden of disease produced to date: the first WHO/ILO Joint Estimates of the Work-related Burden of Disease and Injury (WHO/ILO Joint Estimates).

While it was possible to apply WHO and ILO’s existing methodologies for many established pairs of occupational risk factors and health outcomes, several other pairs were considered in need of a new evidence review. Additionally, some were identified that had not been included in past estimates but were likely to contribute appreciably to the burden of disease. For these potential new pairs, WHO and ILO conducted a series of systematic reviews and meta-analyses of the evidence base, for which protocols were developed, peer-reviewed and pre-published (2–19). These were carried out with support of experts from government departments in 11 countries (often ministries of health and labor) and over 220 individual experts from 35 countries, covering all six WHO regions.

Here we present a brief summary of the methodology and results of the WHO/ILO Joint Estimates. More detail on the burden of disease attributable to 19 selected occupational risk factors (41 pairs of occupational risk factor and health outcome) included in the WHO/ILO Joint Estimates can be found in the WHO/ILO Global Monitoring Report and Technical Report (21). In this article, as in the broader burden of disease framework, the term “burden of disease” refers to the combined burdens of three types of health outcomes, namely communicable diseases, non-communicable diseases and injuries (22, 23).

## Methods

All WHO/ILO Joint Estimates are produced according to the strict statistical rules and established regulations of WHO and the ILO. The data sources and methods used in obtaining these estimates are reported according to the Guidelines for Accurate and Transparent Health Estimates Reporting (GATHER) (24). The technical report and previous publication can be referred to for more details (21, 25).

### Established pairs

Thirty-nine established pairs of occupational risk factors and health outcomes, considered to have a sufficient evidence base, were selected for inclusion (table 1). The burden of disease attributable to established occupational risk factors was estimated using the Comparative Risk Assessment (CRA) framework, a systematic evaluation of the changes in population health that result from modifying the population distribution of exposure to a risk factor or a group of risk factors (22, 23). Recent burden of disease estimates from the Global Burden of Disease Study (26), openly available at http://ghdx.healthdata.org, were used to derive population attributable fractions (PAF). PAF quantify the proportion of deaths or disability-adjusted life years (DALY) lost from a particular health outcome that is attributable to a specific risk factor.

**Table 1:** Total numbers of attributable deaths and disability-adjusted life years (DALY), and numbers of deaths and DALY per 100 000 working-age population (≥15 years) and total population (all ages), by pair of occupational risk factor and health outcome, globally, 183 countries, for 2000, 2010 and 2016. Data source: WHO & ILO (20)

Occupational risk factor ^a^ Health outcome ^b^	Deaths per pair			Deaths per pair per 100 000 population (≥15 years)			DALY per pair			DALY per pair per 100 000 population (≥15 years)		
			
	2000	2010	2016	2000	2010	2016	2000	2010	2016	2000	2010	2016
Established pairs												
Occupational exposure to asbestos												
Trachea, bronchus and lung cancers	137 786	169 697	177 614	3.2	3.4	3.2	2 804 297	3 197 063	3 286 180	65.8	63.4	59.9
Ovary cancer	4519	5214	5464	0.1	0.1	0.1	91 953	99 889	104 297	2.2	2.0	1.9
Larynx cancer	2933	3079	3299	0.1	0.1	0.1	67 006	66 073	69 564	1.6	1.3	1.3
Mesothelioma	12 703	20 567	23 104	0.3	0.4	0.4	327 763	476 621	513 810	7.7	9.4	9.4
Occupational exposure to arsenic												
Trachea, bronchus and lung cancers	5651	6893	7589	0.1	0.1	0.1	183 316	218 684	236 361	4.3	4.3	4.3
Occupational exposure to benzene												
Leukemia	1175	1304	1452	0.0	0.0	0.0	73 681	76 947	85 022	1.7	1.5	1.6
Occupational exposure to beryllium												
Trachea, bronchus and lung cancers	101	138	165	0.0	0.0	0.0	4971	6442	7181	0.1	0.1	0.1
Occupational exposure to cadmium												
Trachea, bronchus and lung cancers	279	392	452	0.0	0.0	0.0	11 696	15 292	17 172	0.3	0.3	0.3
Occupational exposure to chromium												
Trachea, bronchus and lung cancers	620	884	1022	0.0	0.0	0.0	23 888	31 779	36 059	0.6	0.6	0.7
Occupational exposure to diesel engine exhaust												
Trachea, bronchus and lung cancers	9116	12 709	14 728	0.2	0.3	0.3	303 473	410 674	470 650	7.1	8.1	8.6
Occupational exposure to formaldehyde												
Nasopharynx cancer	263	294	327	0.0	0.0	0.0	16 082	16 894	18 056	0.4	0.3	0.3
Leukemia	350	372	416	0.0	0.0	0.0	26 657	26 912	29 143	0.6	0.5	0.5
Occupational exposure to nickel												
Trachea, bronchus and lung cancers	5449	6641	7301	0.1	0.1	0.1	178 881	212 860	229 980	4.2	4.2	4.2
Occupational exposure to polycyclic aromatic hydrocarbons												
Trachea, bronchus and lung cancers	2428	3364	3881	0.1	0.1	0.1	84 081	111 823	126 900	2.0	2.2	2.3
Occupational exposure to silica												
Trachea, bronchus and lung cancers	31 910	38 608	42 258	0.7	0.8	0.8	1 022 981	1 207 501	1 302 917	24.0	23.9	23.8
Occupational exposure to sulfuric acid												
Larynx cancer	2 227	2 303	2 564	0.1	0.0	0.0	81 783	83 960	91 636	1.9	1.7	1.7
Occupational exposure to trichloroethylene												
Kidney cancer	6	18	25	0.0	0.0	0.0	1249	1877	2343	0.0	0.0	0.0
Occupational asthmagens												
Asthma	35 293	30 568	29 641	0.8	0.6	0.5	2 106 628	2 050 770	2 104 429	49.4	40.6	38.4
Occupational particulate matter, gases and fumes												
Chronic obstructive pulmonary disease	473 725	431 992	450 381	11.1	8.6	8.2	11 053 935	10 335 238	10 855 103	259.4	204.8	197.9
Occupational noise												
Other hearing loss	0	0	0	0.0	0.0	0.0	5 917 732	7 280 576	8 164 140	138.9	144.3	148.9
Occupational injuries ^c^												
Pedestrian road injuries	78 790	72 032	72 157	1.8	1.4	1.3	4 547 165	4 214 378	4 244 768	106.7	83.5	77.4
Cyclist road injuries	10 915	10 521	12 018	0.3	0.2	0.2	781 662	802 973	932 514	18.3	15.9	17.0
Motorcyclist road injuries	41 945	44 311	48 151	1.0	0.9	0.9	2 805 094	2 988 019	3 249 277	65.8	59.2	59.2
Motor vehicle road injuries	67 879	70 268	76 946	1.6	1.4	1.4	4 120 501	4 261 916	4 639 833	96.7	84.5	84.6
Other road injuries	1 764	1 807	1 859	0.0	0.0	0.0	172 682	198 907	231 259	4.1	3.9	4.2
Other transport injuries	21 597	17 797	16 864	0.5	0.4	0.3	1 868 380	1 587 934	1 584 940	43.8	31.5	28.9
Poisoning by carbon monoxide	7408	4249	3772	0.2	0.1	0.1	411 082	239 498	213 606	9.6	4.7	3.9
Poisoning by other means	10 477	6313	5330	0.2	0.1	0.1	626 837	389 740	340 195	14.7	7.7	6.2
Falls	36 808	34 064	34 996	0.9	0.7	0.6	3 535 943	3 472 602	3 726 068	83.0	68.8	67.9
Fire, heat and hot substances	16 002	11 342	10 234	0.4	0.2	0.2	1 201 594	946 261	920 655	28.2	18.8	16.8
Drowning	33 135	26 779	26 281	0.8	0.5	0.5	1 956 331	1 559 372	1 530 312	45.9	30.9	27.9
Unintentional firearm injuries	6348	5477	5079	0.1	0.1	0.1	424 086	357 843	344 830	10.0	7.1	6.3
Other exposure to mechanical forces	21 308	18 121	17 406	0.5	0.4	0.3	1 900 679	1 765 361	1 798 106	44.6	35.0	32.8
Pulmonary aspiration and foreign body in airway	8470	7942	7831	0.2	0.2	0.1	420 613	383 236	380 882	9.9	7.6	6.9
Foreign body in other body part	794	635	649	0.0	0.0	0.0	163 163	149 381	165 778	3.8	3.0	3.0
Non-venomous animal contact	1495	1161	1213	0.0	0.0	0.0	153 866	125 943	130 080	3.6	2.5	2.4
Venomous animal contact	9261	6535	6359	0.2	0.1	0.1	647 679	484 024	478 692	15.2	9.6	8.7
Other unintentional injuries	21 478	17 860	16 138	0.5	0.4	0.3	1 812 672	1 600 309	1 528 257	42.5	31.7	27.9
Occupational ergonomic factors												
Back and neck pain	0	0	0	0.0	0.0	0.0	10 214 925	11 342 041	12 267 159	239.7	224.8	223.7
Recently added risk pairs											
Exposure to long working hours												
Ischemic heart disease	244 844	304 200	346 618	7.9	6.0	6.3	7 548 225	9 368 428	10 655 256	177.1	185.7	194.3
Stroke	334 724	366 524	398 306	5.7	7.3	7.3	10 352 978	11 471 221	12 603 247	242.9	227.4	229.8

a Defined as per the Global Burden of Disease Study classification (26).

b Defined as per the burden of disease classification of the WHO Global Health Estimates (27) with the exception of injuries, which are defined as per Global Burden of Disease Study classification (26).

c Throughout this report the term “Occupational injuries” is used as defined by Ezzati et al. (22, 23) to represent an occupational risk factor within the framework of the global Comparative Risk Assessment. This definition differs from that adopted by the 1982 Thirteenth International Conference of Labour Statisticians, and was revised by the 1998 Sixteenth International Conference of Labour Statisticians to mean “any personal injury, disease or death resulting from an occupational accident”.

### Estimation methods

For each pair, the total number of deaths and DALY for each health outcome in the WHO Global Health Estimates total disease envelopes for 2000, 2010 and 2016 (27) were multiplied by the pair’s PAF. This resulted in the estimates of the numbers of deaths and DALY from the health outcome that are attributable to its respective occupational risk factor. For pairs for which there are larger PAF, a larger fraction of the total burden of disease from the health outcome will be estimated.

### Recently added pairs

Following scoping reviews, 16 additional pairs of occupational risk factors and health outcomes were selected, which may contribute substantially to the work-related burden of disease. Systematic reviews and meta-analyses were conducted for these pairs to gather evidence for the WHO/ILO Joint Estimates on the prevalence of exposure to occupational risk factors and on the effect of exposure to these risk factors on health outcomes (2–19). The new methods used (28) have been published in an international academic journal (29). WHO and ILO determined that there were two pairs for which there was sufficient quality and strength of evidence to proceed to burden of disease estimation: exposure to long working hours (defined as working ≥55 hours per week) and the health outcomes of ischemic heart disease and stroke. It should be noted that evaluation of the evidence for some of these pairs is ongoing.

To provide exposure data for these two new pairs, new WHO/ILO databases on exposure to long working hours were developed from data shared by Member States with one or more of WHO, ILO and Eurostat. The databases provided data on the number of workers exposed to long working hours. They used results from 2324 surveys (mostly labor force surveys) from 154 countries, as well as 1742 quarterly datasets of labor force surveys conducted in 46 countries (25).

### Estimation methods

*Prevalence of exposure*. We used an established multilevel model and data from direct exposure measurements provided by the two new WHO/ILO databases to predict the geographical and temporal prevalence of exposure to long working hours (30). As is essential in modelling studies, several modelling assumptions needed to be made. Based on advice from a WHO/ILO technical advisory group and available evidence, an exposure window of ten years, evenly spaced around a lag time of ten years, was agreed upon (ie, to estimate burden of disease in 2016 for example, exposure was modelled for the time window of 2001–2010). The annual prevalence of exposure to long working hours for each year within this window was used in exposure modelling (25). Sensitivity analyses (eg, altering the lag time and the length of the time window) were conducted to test our assumptions.

*Burden of disease*. As for the established risk factors, the burden of disease attributable to exposure to long working hours was estimated within the CRA framework (22, 23). From the systematic reviews, a pooled risk ratio (RR) of 1.17 [95% confidence interval (CI) 1.05–1.31] was found for risk of ischemic heart disease following exposure to long working hours (≥55 hours/week) (9); for stroke, a pooled RR of 1.35 (95% CI 1.13–1.61) was found (3). These pooled RR, along with the prevalence estimates produced by WHO and ILO, were used to calculate the PAF for these two new pairs. These PAF were then applied to the health outcomes’ total disease burden envelopes from the WHO Global Health Estimates for the years 2000, 2010 and 2016 (27), as described above for the established pairs, yielding the number of deaths and DALY from each health outcome attributable to exposure to long working hours (25).

*Inequalities*. To improve workers’ health equity between and within countries, health inequalities in the work-related burden of disease must be monitored. For describing inequalities between regions, sexes and age groups, we used the global number of deaths or DALY per 100 000 working-age (≥15 years) population as the reference. For specific regions, sexes or age groups, we calculated (i) the rate difference: the rate for the specific group minus the reference rate (an absolute inequality measure); and (ii) the rate ratio: the rate for the specific group divided by the reference rate (a relative inequality measure) (31).

*Uncertainty*. All estimates of exposure to occupational risk factors and of burden of disease were produced with their 95% UR (25), using bootstrapping (32). Consistent with previous global health estimates (33–36), the 2.5% and 97.5% quantiles of the random deviates of the exposures were calculated and assigned as the lower and upper limits of the UR, respectively. Here, we present 95% UR for key estimates in the text; however, they are available for all estimates in the online estimates repository (www.who.int/teams/environment-climate-change-and-health/monitoring/who-ilo-joint-estimates).

## Results

### Main findings

Globally in 2016, exposure to the 19 selected occupational risk factors was attributable for an estimated 1 879 890 (95% UR 1 835 140–1 924 640) deaths and 89.72 (95% UR 88.61–90.83) million DALY due to the respective health outcomes.

**Figure 1:**
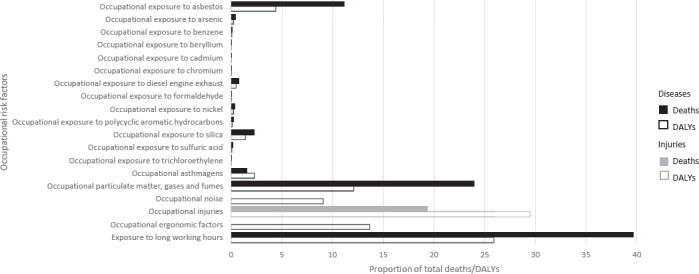
Total number of work-related deaths and DALYs, by occupational risk factor, 183 countries, for the year 2016. Source: WHO and ILO (20).

**Figure 2:**
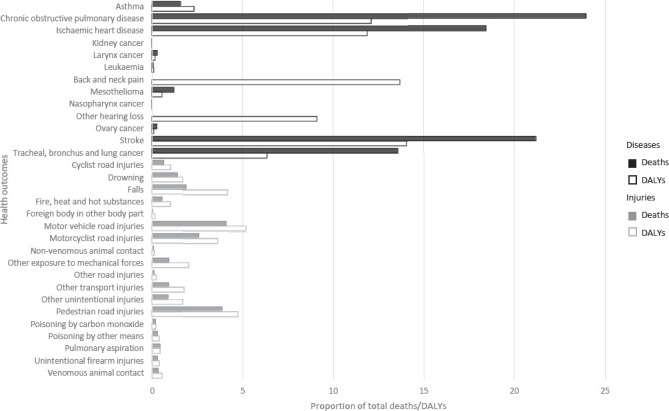
Total number of work-related deaths and DALYs, by health outcome, 183 countries, for the year 2016. Source: WHO and ILO (20).

**Figure 3:**
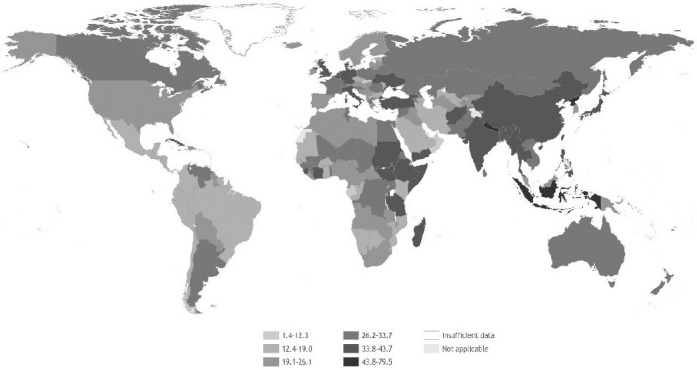
Death rates (per 100 000 working-age population, ie, age ≥15 years) in 2016 from the 41 pairs of occupational risk factors and health outcomes. Source: WHO and ILO (20).

**Figure 4:**
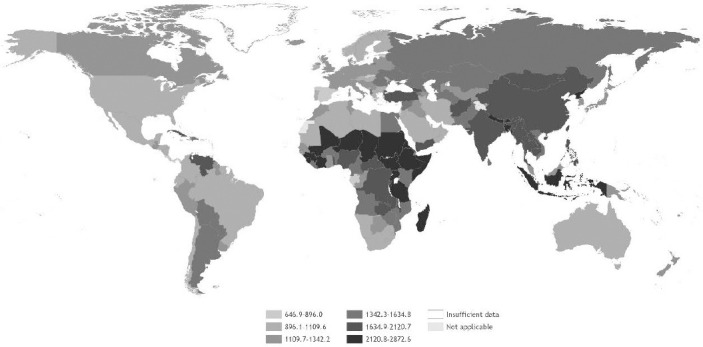
DALY rates (per 100 000 working-age population, ie, age ≥15 years) in 2016 from the 41 pairs of occupational risk factors and health outcomes. Source: WHO and ILO (20).

Table 1 shows the numbers of deaths and DALY corresponding to each occupational risk factor – health outcome pair, with rates per 100 000 working-age population [rates per 100 000 total population are available in the Global Monitoring Report (20)]. The pair with the highest number of deaths was chronic obstructive pulmonary disease attributable to occupational exposure to particulate matter, gases and fumes (450 381 deaths, 95% UR 430 248–470 514). The pair with the highest number of DALY was stroke attributable to exposure to long working hours (12.60 million DALY, 95% CI 11.82–13.39 million). Figure 1 displays deaths and DALY by occupational risk factor and figure 2 displays deaths and DALY by health outcome. Figures 3 and 4 show the rates of deaths and DALY per 100 000 of working-age population by country (numbers provided in the supplementary material, www.sjweh.fi/article/4001, table S1).

The two new pairs of risk factor and health outcome contributed substantially to the burden of work-related disease. Exposure to long working hours and ischemic heart disease, and exposure to long working hours and stroke combined were responsible for 39.6% of deaths (744 924, 95% UR 744 924–784 328) and 25.9% of DALY (23.26 million, 95% UR 22.15–24.37 million), making this previously unquantified occupational risk factor the one with the largest attributable burden of disease. Detailed breakdowns and interpretations of these findings can be found elsewhere (20, 25). Additionally, the WHO/ILO Joint Estimates are available for each pair, disaggregated by sex and age group, and at global, region and country levels, from dedicated websites hosted by WHO (www.who.int/teams/environment-climate-change-and-health/monitoring/who-ilo-joint-estimates) and the ILO (www.ilo.org/global/topics/safety-and-health-at-work/programmes-projects/WCMS_674797/lang--en/index.htm).

### Trends over time

In absolute terms, the global number of occupational risk factor-related deaths from 2000 to 2016 increased by 177 914 between 2000 and 2016. Global work-related DALY increased by 9.67 million from 2000 to 2016.

In terms of rates, globally between 2000 and 2016 rates of total deaths attributable to exposure to occupational risk factors decreased from 39.9 to 34.3 deaths per 100 000 working-age population; this corresponds to a 14.2% decrease in the rate. Similarly, global DALY rates decreased from 1878.4 to 1635.9 DALY per 100 000 working-age population.

### Inequalities in work-related burden of disease

*By geographic region*. The absolute differences in death rates by WHO region, compared with the global rate, ranged from 10.7 (in the South-East Asia Region) to -12.0 deaths per 100 000 working-age population (in the Region of the Americas). For DALY, in absolute terms, rate differences by WHO region ranged from 463.3 DALY per 100 000 working-age population in South-East Asia to -564.1 DALY per 100 000 working-age population in the Americas. For both deaths and DALY, the rate ratios varied from 1.3 for South-East Asia to 0.7 for the Americas.

*By sex*. The death rate per 100 000 working-age males is 51.4, 17.1 per 100 000 higher and 1.5 times the rate for both sexes. The death rate of 17.2 per 100 000 working-age females is lower compared with the rate for both sexes, this is 17.1 per 100 000 lower and 0.5 times this rate. For DALY, a similar pattern was seen.

*By age group*. Older age groups carried disproportionally greater disease burden, with the age group 85–89 years having the highest rate difference (higher than the global rate by 212.6 deaths per 100 000 working-age population) and highest risk ratio (7.2). Conversely, the rate for the age group 15–19 years was 4.3 deaths per 100 000 working-age population, yielding a rate difference of -30.0 and a rate ratio of 0.1 compared to the global rate. Similarly for DALY, the older age groups were more burdened.

## Discussion

WHO and ILO have produced the first set of the WHO/ILO Joint Estimates of the Work-related Burden of Disease and Injury (20). Multiple data sources across all WHO regions have been used to quantify the burden of specific health outcomes attributable to some key occupational risk factors. The WHO/ILO Joint Estimates are available to users disaggregated by sex and age group, at the global, regional and national levels.

Target 8.8 of the SDGs aims to “Protect labour rights and promote safe and secure working environments for all workers, including migrant workers, in particular women migrants, and those in precarious employment”. Although indicator 8.8.1 refers to the “frequency rates of fatal and non-fatal occupational injuries”, injuries accounted for only 19.3% of deaths and 29.5% of DALY attributable to occupational risk factors in 2016. An additional, complementary indicator for Target 8.8, quantifying the burden of deaths from diseases attributable to exposure to occupational risk factors, would more accurately capture the extent of the burden of the work-related disease. The WHO/ILO Joint Estimates can support Member States reporting on indicator 8.8.1 and the proposed indicator, especially where dedicated reporting systems for such deaths may not yet exist.

### Preventive actions

Actions required vary by occupational risk factor, to reduce the burden related to many of the established risk factors and their health impacts (as here quantified), governments should develop interventions to reduce risk factors with the active involvement of employers and workers or their representatives, as part of a hierarchy of controls (39). Where it is not possible to eliminate risk factors or use less hazardous substitutions, engineering controls can be introduced, followed by administrative controls. As a last and least-preferred option, workers can be protected with personal protective equipment.

The burden of stroke and ischemic heart disease attributable to exposure to long working hours was previously unquantified. The generation of the first estimates for this burden may motivate actors to address this risk factor. ILO Conventions define the maximum limits of working hours in industrial and services sectors (40, 41) as 48 hours per week (with some specific exceptions). Human resources management and work organization management can be used to prevent exposure to long working hours, in particular for some specific working modalities (eg, teleworking, self-employment and freelancing) (42). Additionally, occupational health services can play an important role. All workers should be covered (43), and occupational health risk assessments should consider numbers of working hours and other cardiovascular risk factors (eg, obesity, physical activity, smoking and diet) that exposure to long working hours could influence. The introduction of social protection floors would enable people to stop working unhealthy long hours, by guaranteeing access to essential health care and basic income security. This would particularly benefit disadvantaged workers (eg, those in the informal economy, and vulnerable groups such as pregnant women, older people and migrant workers) (44).

### Strengths and limitations

The WHO/ILO Joint Estimates have used established methods to quantify the work-related burden of disease attributable to 19 occupational risk factors (22, 23). A pair was only proceeded for estimation if WHO and ILO judged there to be sufficient quality and strength of evidence for the pair, providing confidence that the burden of disease estimates reported are attributable to the occupational risk factor. As a result, the organizations have estimated that 1.9 million deaths and 89.7 million DALY are attributable to the selected 41 pairs of occupational risk factor and health outcome.

There are some limitations that should be considered when interpreting the estimates. First, these estimates are affected by the source and quality of input data, and the type and complexity of the models of exposure and health estimates. A wide range of approaches have been used to collect and synthesize data. Although estimates have been included only if the underlying body of evidence was judged to be of sufficient quality and strength, some are based on exposure data from limited sources and from areas of limited country and regional coverage. To improve future estimates, more large-scale global official datasets of exposure to occupational risk factors are needed, ideally from direct measurement or through strong proxies such as occupation and industrial sector. Similarly, more primary studies need to be conducted on the effect of exposure to occupational risk factors on health outcomes (29). In particular, more evidence is needed from low- and middle-income countries.

Second, while estimates have been included only if WHO and ILO, supported by a large number of individual experts, judged the underlying body of evidence to be of sufficient quality and strength, this is based on judgement. As this is subjective it may be that other organizations or individuals reach a different judgement. This was demonstrated by a commentary indicating disagreement with the rating of “sufficient evidence for harmfulness” that there is of long working hours with regard to ischemic heart disease (45). The WHO/ILO Working Group, composed of a large number of individual experts, acknowledged and responded to the commentary, elaborating on why the assigned rating is supported by the evidence (46). WHO and the ILO are collaborating on the WHO/ILO Joint Estimates with over 200 individual experts based around the world, to ensure that judgements made represent the views of a diverse and representative expert group.

Third, it must be noted that not all occupational risk factors and attributable burdens of disease have yet been quantified. The production of estimates for some pairs was not possible in this estimation cycle, such as: occupational exposure to biological risk factors and infectious diseases; occupational exposure to psycho-social risk factors and mental health outcomes; and occupational exposure to ambient air pollution and its various health outcomes. Further, while there are established methods for estimating the burdens of silicosis, asbestosis, coal worker’s pneumoconiosis and unspecified pneumoconiosis attributable to occupational exposure to dusts and fibers, WHO and the ILO are currently reviewing these methods and the available bodies of evidence (10); these pairs were therefore not included in this estimation cycle. While this means that the work-related burden of disease is almost certainly higher than the current estimate of selected pairs, the addition of such pairs in future will broaden the scope of these estimates and capture the work-related burden of disease more comprehensively.

Fourth, estimates by their nature are modelled based on certain assumptions (like the appropriate time window of exposure). While the assumptions made draw from the latest evidence base and are transparently reported, as evidence develops, it is possible that new evidence may emerge in the future which could lead us to alter these assumptions. Sensitivity analyses were performed and reported to assess the impact of alternative assumptions related to exposure to long working hours (25).

### Concluding remarks

The WHO/ILO Joint Estimates report that globally in 2016 1.88 million deaths and 89.72 million DALY from health outcomes were estimated to be attributable to the 19 occupational risk factors covered. A disproportionately large work-related burden of disease is observed in the WHO African Region (for DALY), South-East Asia Region and the Western Pacific Region (for deaths), males and older age groups. Future steps should include estimation of disease burden for more occupational risk factor and health outcome pairs, as more high-quality data and evidence become available, to ensure more of the work-related burden of disease is captured.

The WHO/ILO Joint Estimates have widened the scope of the global CRA and strengthened the global capacity for modelling disease burden in occupational health. They allow the global monitoring of exposure to occupational risk factors and the work-related burden of disease, to detect inequalities and trends over time. This will enable policy-makers and institutions to plan, cost, implement and evaluate actions to prevent exposure to occupational risk factors and their attributable burden of disease.

### Disclaimers

The authors alone are responsible for the views expressed in this article and they do not necessarily represent the views, decisions or policies of the institutions with which they are affiliated.

The designations employed and the presentation of the material in this publication do not imply the expression of any opinion whatsoever on the part of WHO concerning the legal status of any country, territory, city or area or of its authorities, or concerning the delimitation of its frontiers or boundaries. Dotted and dashed lines on maps represent approximate border lines for which there may not yet be full agreement.

Terminology used to refer to countries, territories, and areas as well as representation of countries, territories, and areas, including delimitation of frontiers or boundaries, and any direct or indirect attribution of status in this publication follow exclusively the institutional style and practice of WHO. All reasonable precautions have been taken by WHO to verify the information contained in this publication.

However, the published material is being distributed without warranty of any kind, either expressed or implied. The responsibility for the interpretation and use of the material lies with the reader. In no event shall WHO be liable for damages arising from its use.

### Funding sources

Financial support for the preparation of this publication was provided by the United States Centers for Disease Control and Prevention National Institute for Occupational Safety and Health through its cooperative agreement with WHO (grant nos 1E11 OH0010676-02, 6NE11OH010461-02-01 and 5NE11OH010461-03-00); the German Federal Ministry of Health (BMG Germany) under the BMG–WHO Collaborative Programme 2020–2023 (WHO specified award reference 70672); and the Spanish Agency for International Cooperation (AECID) (WHO specified award reference 71208). The European Union also provided financial support to the ILO through the Vision Zero Fund (VZF) project on filling data and knowledge gaps on occupational safety and health in global supply chains, implemented within the framework of the ILO Flagship Programme “Safety + Health for All”. The study sponsors had no role in the study design, collection, analysis and interpretation of the data, writing of the report, or the decision to submit the paper for publication.

## Supplementary material

Supplementary material
