# Supplementary material for: Global, regional and national burden of disease attributable to 19 selected occupational risk factors for 183 countries, 2000–2016: A systematic analysis from the WHO/ILO Joint Estimates of the Work-related Burden of Disease and Injury
Source: Scand J Work Environ Health. 2022 Feb 25;48(2):158–68. doi: 10.5271/sjweh.4001 (PMC9045235; doi:10.5271/sjweh.4001)
Supplement: Supplementary material [file SJWEH-48-158-S001.pdf]

Global, regional and national burden of disease attributable to 19 selected occupational risk factors for 183 countries, 2000–2016: A systematic analysis from the WHO/ILO Joint Estimates of the Work-related Burden of Disease and Injury[1]

by Frank Pega, PhD,[2] Halim Hamzaoui, MD, Bálint Náfrádi, PhD, Natalie C Momen, PhD

1. Supplementary table
2. Corresponding author: Dr Frank Pega, Department of Environment, Climate Change and Health, World Health Organization, Avenue Appia 20, 1211 Geneva 27, Switzerland. [E-mail: pegaf@who.int]

Table S1. Total numbers of attributable deaths and DALYs, and numbers of death and DALY per 100 000 of the working-age population (≥ 15 years), globally and by WHO region and country, 183 countries, for 2000, 2010 and 2016

	No. deaths			No. deaths per 100 000 working- age population (≥ 15 years)			No. DALYs			No. DALYs per 100 000 working-age population (≥ 15 years)		
	2000	2010	2016	2000	2010	2016	2000	2010	2016	2000	2010	2016
Global	1 701 976	1 762 975	1 879 890	39.9	34.9	34.3	80 048 208	83 637 261	89 716 654	1 878.4	1 657.7	1 635.9
African Region	115 547	132 350	150 427	31.2	27.1	25.9	7 962 057	9 462 719	10 846 723	2 147.2	1 935.1	1 867.1
Region of the Americas	163 799	163 549	169 238	27.5	23.4	22.3	7 531 962	77 70 545	8 134 604	1 265.3	1 112.3	1 071.8
South-East Asia Region	503 777	565 746	634 096	48.3	44.6	45.0	24 613 276	26 699 718	29 573 376	2 362.1	2 104.2	2 099.2
European Region	251 306	239 935	229 262	36.2	32.5	30.4	10 709 047	10 081 919	9 631 762	1 543.3	1 365.2	1 276.9
Eastern Mediterranean Region	96 516	111 779	121 725	33.7	28.9	27.2	4 741 158	5 613 679	6 139 952	1 654.3	1 450.6	1 371.7
Western Pacific Region	571 031	549 616	575 142	44.9	37.6	37.5	24 490 708	24 008 682	25 390 237	1 924.0	1 640.6	1 655.5
Afghanistan	5 096	6 334	6 747	48.0	41.9	34.2	289 729	347 281	375 992	2 728.2	2 296.2	1 906.8
Albania	504	543	561	23.1	23.8	23.8	34 351	34 633	33 905	1 575.7	1 515.0	1 439.2
Algeria	5 067	5 160	5 630	24.9	19.7	19.6	264 668	293 440	318 879	1 299.0	1 121.6	1 109.6
Angola	2 906	2 715	3 274	33.6	21.9	21.4	188 819	184 452	219 226	2 181.2	1 491.2	1 435.6
Antigua and Barbuda	2	2	1	3.7	3.0	1.4	476	544	604	878.2	816.8	823.5
Argentina	9 851	9 885	9 478	37.3	32.7	29.1	442 118	453 774	464 847	1 676.2	1 500.2	1 426.1
Armenia	715	716	677	31.4	30.9	28.9	39 014	37 648	37 452	1 713.2	1 624.6	1 599.6
Australia	5 463	5 663	5 812	36.4	31.6	29.6	190 284	200 317	205 576	1 266.4	1 116.7	1 045.4
Austria	1 803	1 814	1 826	26.9	25.3	24.3	76 371	78 109	79 772	1 137.9	1 089.0	1 062.5
Azerbaijan	1 188	1 260	1 280	21.2	18.1	17.1	70 734	86 014	93 144	1 264.8	1 234.1	1 244.7
Bahamas	14	19	20	6.6	7.3	6.9	1 509	1 872	2 075	716.2	721.3	716.3
Bahrain	57	87	96	12.3	8.8	8.5	4 414	10 497	11 549	950.7	1 061.6	1 017.7
Bangladesh	35 180	43 094	49 234	43.7	42.9	43.7	2 113 177	2 320 878	2 613 020	2 625.7	2 312.3	2 321.6
Barbados	33	30	31	15.5	13.2	13.2	1 893	2 043	2 101	891.7	902.3	896.0
Belarus	3 556	2 894	2 162	44.2	36.1	27.4	145 589	126 049	105 603	1 810.2	1 572.2	1 339.2
Belgium	3 736	3 840	3 528	44.1	42.2	37.4	114 448	113 854	108 282	1 350.3	1 252.2	1 149.2

Belize	21	22	27	14.3	10.6	10.7	1 788	2 174	2 687	1 220.1	1 047.1	1 065.1
Benin	796	1 132	1 361	21.1	21.9	21.9	58 280	82 650	96 122	1 547.2	1 600.0	1 547.5
Bhutan	176	190	204	49.5	40.3	37.8	10 552	12 410	13 533	2 966.5	2 630.3	2 507.5
Bolivia (Plurinational State of)	1 717	1 713	1 867	32.8	26.0	24.9	88 875	97 184	105 489	1 695.7	1 476.3	1 405.9
Bosnia and Herzegovina	883	784	670	29.7	25.1	23.3	46 667	40 391	36 453	1 569.0	1 293.6	1 265.3
Botswana	199	248	259	19.7	19.2	18.4	14 343	16 584	17 634	1 422.8	1 283.3	1 250.5
Brazil	29 184	28 594	28 355	23.8	19.4	17.6	1 604 011	1 678 162	1 703 521	1 309.8	1 140.6	1 060.0
Brunei Darussalam	21	21	32	9.1	7.3	10.0	3 260	3 226	3 824	1 411.1	1 121.1	1 196.8
Bulgaria	1 146	1 084	961	17.0	16.9	15.7	63 754	62 655	57 788	945.1	975.5	943.0
Burkina Faso	1 355	1 690	1 869	21.9	20.1	18.4	102 415	133 536	149 784	1 657.5	1 591.2	1 470.9
Burundi	878	1 662	2 067	27.6	34.9	36.2	66 990	127 232	158 553	2 103.4	2 673.3	2 777.0
Cabo Verde	45	47	40	18.4	14.1	10.7	3 217	3 547	3 654	1 317.3	1 063.7	976.9
Cambodia	2 631	2 928	3 179	37.1	30.7	29.4	161 796	184 479	198 659	2 278.9	1 933.2	1 836.5
Cameroon	1 960	2 365	2 874	23.0	20.6	21.1	149 207	182 193	213 018	1 751.5	1 590.6	1 563.7
Canada	8 709	9 077	9 266	35.2	31.8	30.3	343 861	346 419	351 220	1 390.7	1 214.8	1 147.8
Central African Republic	698	680	594	33.8	27.7	23.8	43 286	43 058	42 668	2 096.2	1 754.5	1 708.2
Chad	1 704	2 208	2 536	39.9	36.1	33.3	114 071	151 235	176 585	2 670.2	2 469.8	2 316.0
Chile	1 753	2 118	2 168	15.7	15.9	14.9	104 783	129 468	136 739	939.8	973.8	942.2
China	479 454	442 898	460 257	49.4	39.8	39.7	19 905 168	18 973 358	20 011 944	2 050.7	1 704.1	1 725.8
Colombia	5 874	6 193	6 719	22.0	18.8	18.4	290 039	316 957	336 312	1 085.4	963.7	919.1
Comoros	50	64	80	16.5	15.7	16.8	6 626	8 346	9 624	2 180.7	2 052.5	2 015.3
Congo	413	452	434	22.8	18.1	15.0	29 251	34 088	33 838	1 617.2	1 366.6	1 172.8
Costa Rica	474	500	519	17.4	14.4	13.6	27 303	30 710	31 768	1 001.0	886.4	829.7
Côte d’Ivoire	3 091	4 476	5 158	33.3	38.7	37.6	185 261	264 692	302 549	1 997.4	2 286.5	2 204.1
Croatia	1 001	1 277	1 212	27.3	34.9	33.7	45 154	48 915	44 554	1 233.4	1 336.6	1 237.2
Cuba	3 005	3 446	3 349	34.4	37.3	35.4	147 706	178 810	172 179	1 692.9	1 936.4	1 820.7
Cyprus	155	169	170	21.2	18.5	17.5	7 143	8 383	8 117	975.8	917.0	834.4
Czechia	2 022	1 781	1 707	23.5	19.7	19.0	108 098	103 119	103 276	1 257.0	1 141.2	1 147.0
Democratic People's Republic of Korea	9 540	14 832	15 953	56.2	78.1	79.5	417 288	553 070	576 494	2 457.3	2 912.2	2 872.6
Democratic Republic of the Congo	8 562	11 098	12 179	33.4	31.9	28.8	610 866	801 958	896 504	2 381.2	2 306.2	2 120.7
Denmark	1 828	1 801	1 849	42.0	39.5	38.8	64 261	62 569	63 585	1 475.7	1 372.8	1 334.9
Djibouti	66	85	114	15.6	15.0	17.6	6 737	7 998	9 778	1 590.6	1 411.6	1 510.6
Dominican Republic	1 464	1 626	1 750	26.6	24.2	23.6	84 680	94 187	100 823	1 535.8	1 400.0	1 356.8
Ecuador	1 886	1 753	2 030	22.9	16.9	17.3	112 494	113 496	127 965	1 364.1	1 096.4	1 088.4
Egypt	12 536	17 136	19 599	28.8	30.7	31.3	547 274	767 434	869 717	1 259.2	1 374.9	1 387.8
El Salvador	636	444	476	17.0	10.5	10.4	36 192	27 456	29 665	970.2	649.5	646.9
Equatorial Guinea	118	139	171	32.8	24.0	22.5	8 506	10 055	11 731	2 364.2	1 732.7	1 543.3
Eritrea	556	636	637	44.7	33.1	32.6	33 555	41 093	42 895	2 696.2	2 141.8	2 192.7
Estonia	245	180	154	21.2	15.9	13.9	14 121	10 865	9 790	1 224.6	961.1	886.7
Eswatini	91	99	100	15.9	15.5	14.6	6 097	5 950	6 811	1 064.0	934.2	996.5
Ethiopia	14 504	17 343	21 841	40.9	35.9	36.2	1 090 203	1 335 548	1 676 235	3 074.9	2 767.3	2 776.2
Fiji	158	138	131	30.0	22.6	21.4	9 082	8 445	8 120	1 723.7	1 383.4	1 324.8
Finland	1 076	1 124	1 089	25.3	25.1	23.7	45 244	44 577	44 022	1 065.3	995.2	957.1
France	14 321	16 232	17 209	29.9	31.7	32.6	577 533	648 275	661 506	1 206.8	1 264.7	1 251.2

Gabon	129	129	146	17.8	12.7	11.5	8 492	9 629	10 694	1 172.1	947.6	839.9
Gambia	196	217	235	28.1	22.1	19.7	13 523	14 863	16 361	1 939.0	1 511.0	1 370.5
Georgia	1 333	1 448	1 397	38.6	43.1	43.1	68 165	64 079	56 683	1 973.6	1 905.4	1 748.2
Germany	22 811	23 728	24 294	33.2	34.0	34.1	881 259	857 368	867 764	1 283.8	1 227.3	1 217.9
Ghana	1 988	2 744	3 028	17.9	18.2	17.2	137 257	176 780	201 021	1 237.2	1 175.0	1 138.8
Greece	2 561	2 462	2 384	27.2	26.6	26.2	108 150	100 552	94 420	1 149.2	1 087.2	1 038.6
Grenada	7	5	5	10.2	6.2	5.9	651	664	660	950.4	822.1	779.7
Guatemala	1 556	1 599	1 804	23.7	18.0	16.9	105 088	108 102	122 161	1 603.0	1 218.6	1 144.9
Guinea	1 666	2 007	2 094	37.7	36.6	32.2	118 620	140 298	148 097	2 687.2	2 557.4	2 279.1
Guinea-Bissau	125	127	132	19.1	14.7	12.9	10 208	11 539	12 779	1 556.7	1 333.0	1 249.0
Guyana	104	110	128	21.6	21.7	23.4	6 206	6 314	7 112	1 291.6	1 242.9	1 297.9
Haiti	1 370	1 314	1 817	27.1	20.7	25.4	62 962	68 735	86 877	1 244.9	1 084.0	1 214.4
Honduras	593	721	964	15.8	13.9	15.5	39 784	49 401	63 060	1 058.3	951.5	1 015.2
Hungary	1 951	1 840	1 897	23.0	21.8	22.7	94 303	87 722	90 360	1 109.4	1 037.9	1 082.0
Iceland	49	63	57	22.8	24.8	21.5	2 965	3 075	3 036	1 376.7	1 212.0	1 144.4
India	345 418	370 599	416 910	50.1	43.4	43.7	16 285 249	16 893 968	18 850 632	2 361.3	1 978.2	1 974.8
Indonesia	73 328	90 778	102 523	50.0	52.7	53.9	3 678 072	4 505 634	4 985 614	2 509.0	2 617.8	2 619.5
Iran (Islamic Republic of)	11 557	12 038	11 303	26.7	21.5	18.7	551 469	574 026	563 881	1 272.6	1 024.6	932.9
Iraq	2 927	3 644	4 486	21.8	21.1	20.1	142 952	200 919	264 512	1 066.0	1 160.8	1 186.4
Ireland	960	748	808	32.3	20.7	22.0	35 044	33 707	35 134	1 179.5	934.3	956.2
Israel	659	679	716	15.4	12.7	12.3	32 912	39 016	42 289	769.6	730.2	723.6
Italy	18 476	20 058	20 010	38.0	39.4	38.2	618 868	617 513	613 696	1 274.1	1 211.5	1 171.0
Jamaica	390	307	323	21.7	15.0	14.7	20 523	17 788	18 408	1 139.4	867.2	836.8
Japan	33 115	37 853	38 439	30.5	34.0	34.5	1 461 835	1 411 556	1 373 425	1 345.2	1 267.4	1 234.4
Jordan	656	878	1 172	21.2	19.3	19.0	32 364	44 998	58 021	1 046.3	991.1	941.0
Kazakhstan	4 818	4 380	4 011	44.5	35.5	30.9	230 428	218 932	211 893	2 130.6	1 773.8	1 634.8
Kenya	3 575	3 008	3 845	20.4	12.7	13.3	329 257	328 532	406 006	1 881.3	1 382.0	1 400.6
Kiribati	3	5	9	5.9	7.6	12.3	649	830	923	1 281.1	1 262.0	1 260.1
Kuwait	225	405	626	15.4	17.6	20.1	17 174	28 599	41 028	1 172.5	1 244.9	1 317.5
Kyrgyzstan	1 027	1 022	976	32.1	26.9	23.6	51 052	54 290	55 493	1 594.7	1 428.2	1 341.9
Lao People's Democratic Republic	1 239	1 409	1 504	41.1	35.5	32.9	64 532	73 471	80 643	2 140.1	1 849.5	1 763.6
Latvia	751	553	411	38.3	30.4	24.6	33 369	25 458	20 663	1 703.9	1 398.5	1 234.6
Lebanon	768	958	1 277	29.0	26.1	26.1	34 959	43 180	58 528	1 320.3	1 175.2	1 194.0
Lesotho	270	284	288	22.1	21.9	20.8	14 086	12 915	13 671	1 155.1	993.9	986.6
Liberia	434	595	627	26.6	26.8	23.5	29 410	38 854	43 262	1 801.7	1 752.8	1 620.7
Libya	804	877	899	22.7	19.9	19.4	39 482	43 334	43 869	1 114.0	981.4	948.0
Lithuania	861	764	589	30.7	28.7	23.9	41 581	35 348	29 284	1 485.0	1 327.6	1 187.3
Luxembourg	88	94	96	24.9	22.5	19.8	3 861	3 997	4 323	1 092.3	955.6	891.0
Madagascar	4 240	5 504	5 572	49.0	46.1	38.1	271 668	371 771	391 327	3 141.5	3 112.1	2 677.7
Malawi	1 962	1 666	1 954	32.6	21.4	20.5	140 849	138 567	164 077	2 343.0	1 776.4	1 724.0
Malaysia	3 948	4 491	4 982	25.5	22.1	21.6	207 414	242 120	268 837	1 341.8	1 191.5	1 163.8
Maldives	43	37	36	25.9	13.5	9.6	2 420	2 427	2 598	1 454.9	888.3	689.2
Mali	2 097	2 545	2 915	35.9	32.2	31.1	134 726	176 769	202 754	2 303.4	2 236.8	2 164.3
Malta	66	75	95	20.9	21.3	25.4	3 508	3 846	4 039	1 111.9	1 092.1	1 079.6
Mauritania	223	297	369	15.0	14.5	14.9	17 095	23 142	26 794	1 151.2	1 127.3	1 079.9
Mauritius	170	136	170	19.3	14.0	16.6	9 277	8 824	9 772	1 054.3	905.9	954.9

Mexico	13 364	15 570	16 452	20.5	19.4	18.3	766 404	833 132	892 709	1 177.6	1 035.9	995.7
Micronesia (Federated States of)	2	6	5	3.1	9.1	6.7	873	786	807	1 362.7	1 187.5	1 082.0
Mongolia	612	665	715	39.1	33.5	33.2	30 406	36 217	39 180	1 944.4	1 824.6	1 816.6
Montenegro	84	80	73	17.4	15.9	14.3	5 447	4 959	4 861	1 130.1	983.6	948.9
Morocco	6 619	5 046	4 933	34.6	21.8	19.4	336 180	302 909	304 496	1 755.4	1 310.1	1 198.0
Mozambique	3 882	2 912	2 741	39.5	22.8	18.0	272 514	238 726	242 743	2 772.4	1 869.4	1 592.1
Myanmar	10 606	13 456	15 140	33.6	38.0	39.3	505 414	636 153	707 994	1 603.0	1 797.2	1 836.7
Namibia	181	180	197	17.4	13.6	13.2	11 141	12 703	14 229	1 070.8	957.9	955.9
Nepal	9 185	9 500	9 726	65.0	55.2	52.7	492 447	466 409	472 163	3 484.2	2 710.0	2 558.2
Netherlands	5 589	5 506	5 621	43.0	40.0	39.7	181 011	178 240	179 292	1 394.0	1 295.4	1 265.6
New Zealand	1 036	1 051	1 016	34.7	30.3	27.2	41 775	43 539	43 601	1 400.7	1 253.2	1 167.0
Nicaragua	527	576	633	17.2	14.8	14.5	33 912	39 876	43 902	1 108.6	1 026.8	1 007.9
Niger	1 872	3 106	3 457	31.9	37.8	33.4	137 139	216 112	241 770	2 339.8	2 627.2	2 333.3
Nigeria	18 343	21 279	24 461	26.6	24.0	23.5	1 330 528	1 558 318	1 757 639	1 929.3	1 756.7	1 691.0
North Macedonia	290	288	281	18.4	16.9	16.2	16 147	17 124	17 069	1 026.3	1 007.3	985.7
Norway	935	1 053	1 006	26.0	26.5	23.3	38 140	40 849	40 092	1 059.4	1 029.8	928.9
Oman	261	300	418	18.3	13.3	11.9	15 463	19 637	32 168	1 084.0	868.8	919.1
Pakistan	32 481	37 237	41 045	39.3	33.3	31.3	1 497 441	1 715 934	1 890 025	1 812.6	1 535.0	1 443.4
Panama	324	346	352	15.7	13.4	12.0	20 525	21 531	22 392	995.8	834.0	766.1
Papua New Guinea	878	966	1 257	24.9	21.4	23.9	48 553	55 406	67 829	1 378.4	1 228.4	1 290.6
Paraguay	938	976	1 078	28.6	23.3	22.7	57 839	60 871	66 917	1 766.0	1 452.7	1 410.9
Peru	3 570	3 856	4 250	20.6	19.0	18.9	196 057	229 906	256 021	1 130.2	1 133.0	1 135.6
Philippines	13 577	22 168	26 635	28.3	35.7	37.7	784 393	1 108 527	1 286 094	1 634.5	1 787.2	1 822.4
Poland	7 454	8 637	9 174	24.0	26.6	28.3	395 050	408 363	412 287	1 273.7	1 256.6	1 273.6
Portugal	2 643	2 159	1 910	30.6	24.0	21.5	116 466	102 759	96 018	1 347.0	1 140.5	1 079.7
Qatar	40	138	227	9.1	8.5	9.9	4 792	16 354	22 616	1 088.9	1 011.3	985.0
Republic of Korea	11 345	9 642	9 105	30.2	23.2	20.6	658 929	606 062	591 949	1 751.9	1 458.0	1 342.2
Republic of Moldova	851	792	602	26.5	23.2	17.6	45 382	40 485	34 682	1 410.7	1 186.8	1012.9
Romania	7 011	5 586	4 754	38.9	32.4	28.4	349 360	269 904	239 628	1 938.5	1 565.4	1 431.2
Russian Federation	51 714	40 001	33 976	43.2	32.8	28.3	2 349 918	1 942 360	1 749 394	1 963.3	1 591.3	1 455.9
Rwanda	1 466	1 262	1 450	33.2	21.5	20.8	110 101	113 349	131 244	2 494.8	1 931.3	1 884.7
Saint Lucia	15	11	14	14.1	8.2	9.6	1 072	1 209	1 330	1 010.1	904.4	915.8
Saint Vincent and the Grenadines	2	6	8	2.7	7.5	9.5	501	637	732	678.4	793.3	872.5
Samoa	20	16	13	19.4	13.9	10.9	1 790	1 423	1 340	1 731.9	1 240.5	1 122.8
Sao Tome and Principe	5	3	4	6.3	2.9	3.5	876	1 067	1 231	1 106.9	1 045.2	1 065.2
Saudi Arabia	2 614	3 209	3 866	20.5	16.6	16.0	143 477	185 020	221 468	1 124.8	959.8	916.6
Senegal	1 211	1 490	1 503	22.4	20.8	17.7	82 072	104 392	112 013	1 515.0	1 457.5	1 319.4
Serbia	2 464	2 128	1 873	32.7	28.6	25.2	115 143	100 454	91 331	1 526.5	1 351.5	1 227.7
Seychelles	0	0	4	0.0	0.0	5.5	727	886	899	1 257.9	1 257.6	1 224.9
Sierra Leone	1 562	1 631	1 710	61.0	44.6	40.0	80 382	89 275	95 246	3 140.4	2 442.9	2 226.7
Singapore	788	776	803	24.1	17.6	16.2	36 530	37 665	39 081	1 115.4	853.5	788.1
Slovakia	569	520	506	13.1	11.4	11.0	37 008	36 659	36 405	853.7	801.2	789.4
Slovenia	481	509	496	28.7	29.0	28.1	22 398	23 137	22 509	1 337.8	1 316.5	1 273.4
Solomon Islands	56	57	62	23.4	18.2	16.8	3 665	3 613	3 869	1 529.2	1 155.2	1 048.5
Somalia	1 820	2 213	2 667	38.9	35.5	35.4	114 122	148 404	174 268	2 436.1	2 377.6	2 315.4

South Africa	7 344	7 516	7 977	24.7	20.9	20.0	361 163	380 297	411 807	1 214.4	1 055.8	1 035.0
South Sudan	1 641	1 930	2 296	48.0	35.9	36.7	115 748	146 498	177 351	3 383.8	2 727.8	2 836.2
Spain	9 005	9 320	9 186	25.9	23.3	23.1	345 474	364 338	348 157	992.5	911.1	875.9
Sri Lanka	3 933	4 077	4 666	28.6	27.0	29.4	198 443	214 853	236 553	1 442.8	1 421.0	1 492.3
Sudan	8 071	8 536	9 259	52.7	43.3	39.5	448 734	475 962	508 866	2 927.5	2 417.0	2 171.4
Suriname	72	66	74	22.7	17.7	18.1	3 873	3 861	4 210	1 222.1	1 033.6	1 030.6
Sweden	1 853	1 850	1 856	25.6	23.6	22.8	77 370	77 631	78 147	1 067.8	990.2	961.7
Switzerland	1 853	1 881	1 893	31.4	28.4	26.5	79 550	83 653	84 745	1 348.8	1 261.2	1 187.4
Syrian Arab Republic	3 067	4 084	3 546	31.7	30.5	30.0	166 867	223 873	184 005	1 723.8	1 673.8	1 557.7
Tajikistan	841	924	1 101	23.5	19.1	19.9	51 171	57 119	70 297	1 432.2	1 179.7	1 270.4
Thailand	16 215	19 042	19 559	33.9	35.1	34.4	899 204	1 083 594	1 104 180	1 879.0	1 995.4	1 943.9
Timor-Leste	153	141	145	31.4	22.4	19.5	11 009	10 321	10 595	2 258.1	1 642.4	1 421.4
Togo	1 062	1 261	1 382	38.0	34.3	31.7	61 552	74 924	84 386	2 202.5	2 039.3	1 934.2
Tonga	5	4	5	8.3	6.1	7.7	812	732	734	1 347.5	1 124.7	1 136.2
Trinidad and Tobago	199	203	203	21.1	19.3	18.6	9 192	9 914	10 223	974.9	941.6	934.9
Tunisia	2 101	2 321	2 361	30.7	28.5	27.5	87 766	97 161	100 970	1 283.3	1 191.6	1 175.3
Turkey	22 110	23 255	22 438	50.4	44.0	37.6	1 136 939	1 083 095	1 010 894	2 590.6	2 048.6	1 695.8
Turkmenistan	627	679	767	21.8	18.9	19.6	42 365	50 018	53 227	1 471.9	1 395.0	1 359.0
Uganda	4 229	4 994	5 843	35.5	30.3	28.2	312 947	382 876	453 116	2 628.6	2 319.3	2 184.0
Ukraine	20 080	16 252	12 978	49.6	41.3	34.3	794 020	663 706	532 841	1 961.6	1 687.8	1 409.2
United Arab Emirates	305	687	839	13.2	9.3	10.5	24 716	65 399	76 116	1 066.3	880.8	950.2
United Kingdom	20 963	21 905	22 686	43.9	41.8	41.5	638 095	650 738	663 041	1 337.5	1 242.9	1 213.6
United Republic of Tanzania	9 059	9 758	11 319	49.0	39.9	38.4	646 712	719 517	822 583	3 495.3	2 943.3	2 794.3
United States of America	68 695	64 900	67 198	31.1	26.3	25.7	2 505 083	2 426 189	2 525 864	1 135.7	984.0	965.7
Uruguay	817	790	740	32.6	30.2	27.3	34 148	35 249	34 356	1 363.1	1 348.8	1 269.1
Uzbekistan	3 299	3 197	3 289	21.2	15.8	14.6	214 646	235 370	250 523	1 381.4	1 164.8	1 112.7
Vanuatu	15	19	22	13.9	13.0	12.9	2 019	2 341	2 685	1 866.0	1 604.0	1 578.8
Venezuela (Bolivarian Republic of)	6 633	6 771	7 139	41.5	34.0	33.3	380 412	383 911	409 675	2 381.6	1 925.9	1 911.9
Viet Nam	16 665	18 840	21 159	30.5	28.0	29.4	876 944	1 014 568	1 161 117	1 603.8	1 510.1	1 611.1
Yemen	4 445	5 566	6 245	50.0	41.9	38.5	235 044	294 758	328 078	2 643.7	2 220.6	2 024.9
Zambia	1 635	1 772	1 849	29.3	24.7	20.9	119 941	138 824	153 483	2 148.7	1 935.7	1 731.8
Zimbabwe	1 987	1 783	1 755	28.9	24.0	21.8	108 383	112 813	124 057	1 574.5	1 521.6	1 542.1

DALYs, disability-adjusted life years.
